# Comparison of Torque Teno Virus DNA Load in Plasma and Whole Blood

**DOI:** 10.1016/j.ekir.2025.04.033

**Published:** 2025-04-22

**Authors:** Aurélie Truffot, Lara Cabezas, Manon Gnesotto, Julien Lupo, Patrice Morand, Thomas Jouve, Johan Noble, Raphaële Germi

**Affiliations:** 1 Univ. Grenoble Alpes, CEA, CNRS, CAID IBS, CHU Grenoble Alpes, Laboratoire de Virologie, Grenoble, France; 2 Univ. Grenoble Alpes, IAB, CNRS, Inserm, CHU Grenoble Alpes, Service de Néphrologie, Grenoble, France

**Keywords:** kidney transplantation, opportunistic infections, plasma, Torque teno virus, whole blood

## Introduction

The main challenge of immunosuppressive therapy after solid organ transplantation is creating an immunological balance that prevents organ rejection without promoting opportunistic infections. Torque teno virus (TTV) is a prevalent, nonpathogenic and ubiquitous DNA virus from the *Anelloviridae* family in humans.^1^ Its replicates in the bone marrow, liver, and T lymphocytes.^2^ Several studies have explored TTV DNA load as a marker of immune function to assess infection and rejection risk in kidney transplant recipients.^3^ TTV DNA load fluctuates and may be influenced by age, immune status, and coinfections.^4^ Our recent work showed that switching immunosuppression from tacrolimus to belatacept did not affect TTV DNA load in whole blood (WB).^5^

Most studies on TTV DNA load have been conducted in plasma,^3^^,^^6^ though the main site of TTV replication is T lymphocytes. WB is widely used in Europe for monitoring Epstein-Barr virus, cytomegalovirus, and polyomavirus BK replication in transplant recipients.^7^ An assay was developed for both plasma and WB.^8^ Uniformity in sample selection for viral monitoring is essential for studies comparisons, which is why we compared TTV DNA loads between paired plasma and WB samples.

The Grenoble University Hospital review board approved this retrospective monocentric study (registration RnIPH 2023, protocol SwitchAbela; CNIL number: 2205066 v0). All patients provided written informed consent for the conservation and reuse of their samples.

## Results

### TTV DNA Status in Plasma and in WB Samples

We performed 218 and 261 TTV quantitative real-time polymerase chain reaction (PCR) assays on plasma and WB, respectively, from kidney transplant patients (Supplementary Methods). The overall prevalence was 92%.

TTV DNA status in the paired plasma and WB samples (216) collected from 68 patients is detailed in Table 1. The Cohen’s kappa concordance test showed substantial agreement between plasma and WB assay for TTV detection (k = 0.616, *P* = 0.098, concordance = 94%).S1 The positive concordance was 88.4%, whereas the negative concordance was 5.5% (Table 1). Samples where TTV DNA was detected only in plasma had low viral loads (1.6–2.8 log copies/ml) and those detected only in WB ranged from 2.4 to 3.1 log copies/ml.

**Table 1:** Concordance in the detection of TTV DNA in plasma and whole blood

	Plasma			Kappa	
	Positive *n* (%)	Negative *n* (%)	Total	K^a^	*P*^b^
Whole blood					
Positive	191 (88.4)	6 (2.8)	197 (91.2)	0.616	0.098
Negative	7 (3.2)	12 (5.5)	19 (8.8)		
Total	198 (91.7)	18 (8.3)	216 (100)		

TTV, Torque teno virus.

a Kappa value.

b Statistical significance if *P* < 0.05.

### Comparison of the TTV DNA Load in Plasma and WB Samples

**Figure 1:**
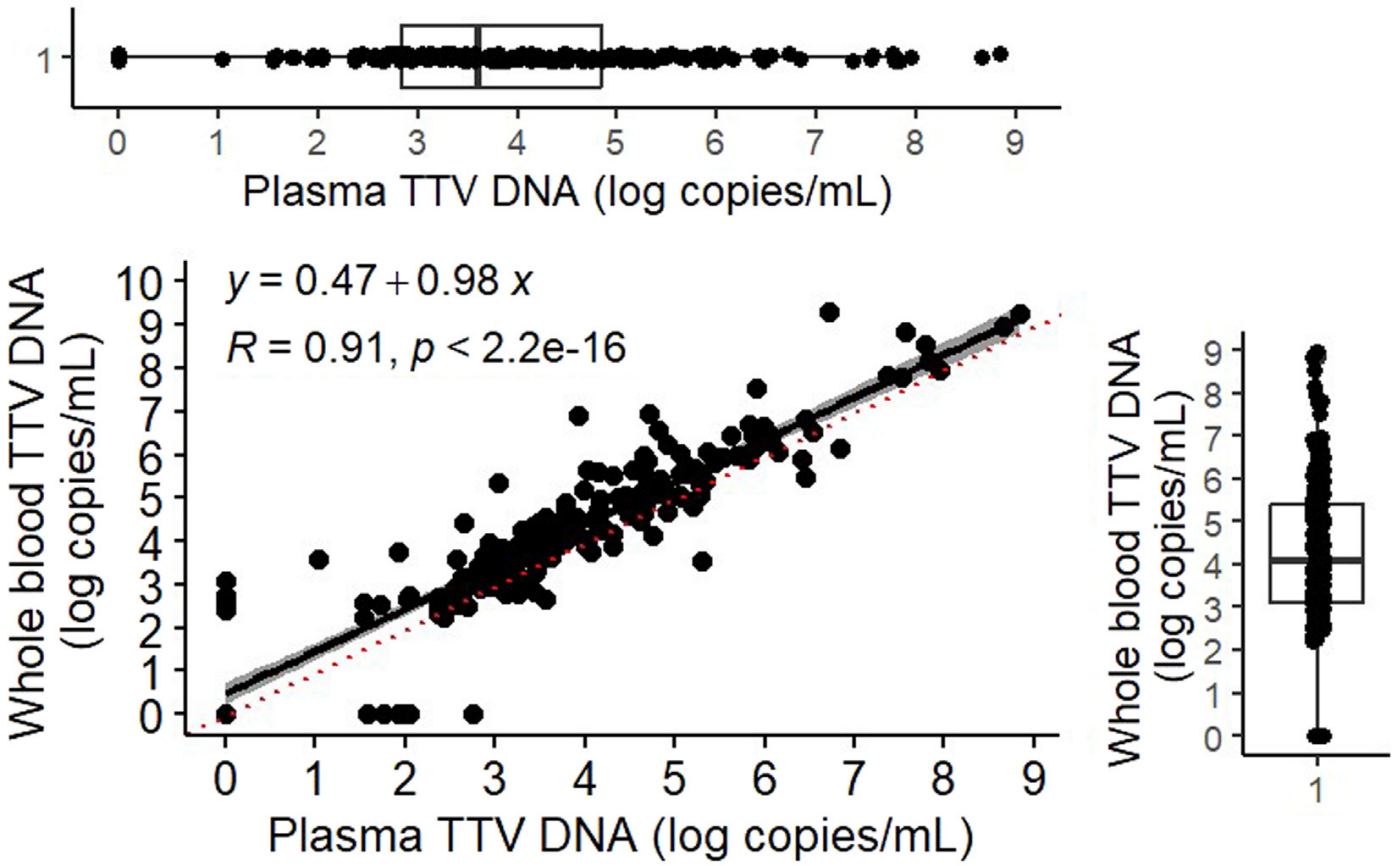
TTV DNA load in plasma and whole blood from paired clinical samples. TTV DNA load in whole blood is plotted as a function of plasma TTV DNA load. Plain line indicates the observed correlation (n = 216; Pearson correlation coefficient = 0.91, P < 0.05). Box plots represent the variability of the TTV DNA load in whole blood (vertical) and in plasma (horizontal). Boxes represent the first quartile (Q1 = 25%) and the third quartile (Q3 = 75%) and central bars represent medians. TTV, Torque teno virus.

The median plasma TTV DNA load (25th–75th percentiles) was 3.7 (2.8–4.8) log copies/ml, whereas the TTV DNA load in WB had a median of 4.1 (3.1, 5.4) log copies/ml (boxplots, Figure 1). The TTV DNA load was statistically higher in WB than in plasma (Mann-Whitney test, *P* = 0.016). The distribution of TTV DNA loads was not significantly different between the 2 matrices (F test, *P* = 0.331). The median difference in TTV DNA load between WB and plasma (TTV DNA load in WB−plasma TTV DNA load) was 3.5 (0–3.1) log copies/ml (Figure 1). The Bland-Altman analysis showed that the mean of the differences obtained between plasma and WB viral load was −0.4 log copies/ml (± 1.96 SD = 1.2 and −2) (Supplementary Figure S1). Nineteen points (8.8%) were considered as outlier.

Paired TTV DNA load in plasma and WB were highly correlated (Pearson’s correlation, R = 0.91 and *P* < 0.001), with a linear regression of TTV DNA load in WB = 0 .47 + 0.98 plasma TTV DNA load (both in log copies/ml, R = 0.9) (Figure 1).

### Epidemiologic Determinants of TTV DNA Variability

Just before immunosuppressive treatment conversion, patients had a median age of 59.6 years and were 55.9% male. All were on tacrolimus and had received a kidney graft over 5 years prior (median: 5.3 years). TTV DNA load was similar between men and women, regardless the matrix (*P* = 0.899 in WB, *P* = 0.118 in plasma). WB TTV DNA load was not correlated with age (R = 0.2) or lymphocytes count (plasma: R = 0.084, *P* = 0.22; WB: R = 0.041, *P* = 0.55).

### Influence of the Immunosuppressive Drug on TTV DNA Load

Plasma and WB TTV DNA loads were explored in 68 kidney-transplant patients who were converted from tacrolimus to belatacept during the study period. Whether on tacrolimus or belatacept, the TTV DNA load was higher in WB than in plasma (Wilcoxon test, *P* = 0.07 and *P* = 0.09 when patients were treated with tacrolimus or belatacept, respectively). Moreover, the switch from tacrolimus to belatacept had no impact on TTV DNA load in either plasma or WB (Wilcoxon test, *P* = 0.30 and *P* = 0.54, respectively) (Supplementary Figure S2).

Four patients had negative TTV quantitative real-time PCR before the switch but tested positive for TTV quantitative real-time PCR 12 months after switching to belatacept, with individual viral load values of 2.2, 3.1, 5.1, and 5.3 log copies/ml.

## Discussion

This is the first study comparing TTV DNA load in WB and plasma in kidney-transplant patients. WB is the preferred matrix in Europe for monitoring viral loads in immunocompromised patients. TTV DNA load was slightly higher in WB (+ 0.47 log copies/ml) than in plasma (*P* < 0.001), with a significant positive correlation (R = 0.9, *P* < 0.001). Two studies have already carried out this comparison but both were based on healthy subjects. Focosi *et al.*^9^ showed on 100 healthy volunteer’s that TTV load in WB was 1.4 log copies/ml higher than in plasma; whereas Kulifaj *et al.*^8^ study on 23 kidney donors or healthy volunteers showed a mean increase of only 0.27 log copies/ml in WB, close to the result of our current study.^9^ This aligns with the findings of Maggi *et al.*,S2 suggesting that TTV replicates in hematopoietic cells and mainly in T lymphocytes. Given the higher TTV load in WB, one might expect more frequent TTV quantitative real-time PCR positive in WB, enabling broader patient monitoring.

A higher TTV DNA load in WB may indicate that the distribution of TTV DNA loads in a population might be broader using this matrix. This would make it easier to observe differences in TTV DNA loads, enhancing its role as a biomarker. However, our finding shows that TTV DNA load in WB is not more effective than in plasma, because the distribution of TTV DNA loads did not differ significantly between the 2 matrices (F test, *P* = 0.331) (Figure 1).

Despite the slightly higher viral load in WB, its use did not enhance TTV detection overall. The concordance between WB and plasma was high (94%). In some cases, TTV was detected in only 1 matrix, likely because of differences in sample processing or low-level viremia near detection limits. Moreover, extraction yields can be different in WB and plasma, which are different matrices, one highly cellular and the other not. Differences in extraction quality could have a major impact on samples with a viral load at the detection limit. Only 1 sample had a viral load of 1150 copies/ml, whereas the rest were below the quantification limit (< 500 copies/ml). The variability in the distribution of the difference between the log copies/ml in WB and the log copies/ml in plasma can be attributed to the fact that some patients exhibit a negative viral load in one matrix but positive in the other (Figure 1).

To date, this potential predictive value has been assessed in patients on tacrolimus. We recently showed that conversion from tacrolimus to belatacept did not affect TTV DNA load when measured in WB.^5^ This suggest that the threshold values for TTV load in tacrolimus-treated patients evaluated in the TTVguideIT study could be applied to patients on belatacept.^S3,S4^ Given the correlation between WB and plasma (TTV DNA load in WB = 0.47 + 0.98 plasma TTV DNA load), the findings from Cabezas *et al.*^5^ on WB could apply to TTV DNA load measured in plasma. Many laboratories are working on WB for cytomegalovirus or Epstein-Barr virus PCR; that is why setting up a conversion factor between the matrices could be interesting.

## Disclosure

All the authors declared no competing interests.

## Supplementary Material

Supplementary File (PDF) **Supplementary Methods. Supplementary References. Figure S1.** Bland-Altman representation of TTV viral loads obtained in plasma and whole blood. **Figure S2.** TTV DNA load performed at the same time in plasma and whole blood of patients on tacrolimus and then 12 months after conversion to belatacept.

## Footnotes

Supplementary File (PDF)

**Supplementary Methods.**

**Supplementary References.**
**Figure S1.** Bland-Altman representation of TTV viral loads obtained in plasma and whole blood. Horizontal lines are drawn at the mean difference (red line), and at the upper and lower limits of agreement (dashed lines). Outliers are presented in red. TTV, torque teno virus. **Figure S2.** TTV DNA load performed at the same time in plasma and whole blood of patients on tacrolimus and then 12 months after conversion to belatacept.

## References

[1] Rezahosseini O., Drabe C.H., Sørensen S.S. (2019). Torque-Teno virus viral load as a potential endogenous marker of immune function in solid organ transplantation. Transplant Rev (Orlando).

[2] Mafi S., Essig M., Rerolle J.P. (2023). Torque Teno virus viremia and QuantiFERON®-CMV assay in prediction of cytomegalovirus reactivation in R+ kidney transplant recipients. Front Med (Lausanne).

[3] van Rijn A., Roos R., Dekker F., Rotmans J., Feltkamp M. (2023). Torque Teno virus load as marker of rejection and infection in solid organ transplantation–a systematic review and meta-analysis. Rev Med Virol.

[4] Focosi D., Antonelli G., Pistello M., Maggi F. (2016). Torquetenovirus: the human virome from bench to bedside. Clin Microbiol Infect.

[5] Cabezas L., Truffot A., Germi R. (2024). Evaluation of Torque Teno virus DNA load as a predictive biomarker in kidney transplant recipients converted from calcineurin inhibitors to Belatacept. Kidney Int Rep.

[6] Batista A.M., Caetano M.W., Stincarelli M.A. (2022). Quantification of torque Teno virus (TTV) DNA in saliva and plasma samples in patients at short time before and after kidney transplantation. J Oral Microbiol.

[7] Lazzarotto T., Chiereghin A., Piralla A. (2018). Cytomegalovirus and Epstein-Barr virus DNA kinetics in whole blood and plasma of allogeneic hematopoietic stem cell transplantation recipients. Biol Blood Marrow Transplant.

[8] Kulifaj D., Durgueil-Lariviere B., Meynier F. (2018). Development of a standardized real time PCR for Torque Teno viruses (TTV) viral load detection and quantification: a new tool for immune monitoring. J Clin Virol.

[9] Focosi D., Spezia P.G., Macera L. (2020). Assessment of prevalence and load of torquetenovirus viraemia in a large cohort of healthy blood donors. Clin Microbiol Infect.

