# Supplementary material for: Comparison of Torque Teno Virus DNA Load in Plasma and Whole Blood
Source: Kidney Int Rep. 2025 Apr 22;10(7):2446–9. doi: 10.1016/j.ekir.2025.04.033 (PMC12266200; doi:10.1016/j.ekir.2025.04.033)
Supplement: Supplementary File (PDF) — Supplementary Methods. Supplementary References. Figure S1. Bland-Altman representation of TTV viral loads obtained in plasma and whole blood. Figure S2. TTV DNA load performed at the same time in plasma and whole blood of patients on tacrolimus and then 12 months after conversion to belatacept. [file mmc1.pdf]

SUPPLEMENTARY MATERIAL

Comparison of Torque teno virus DNA load in plasma and whole blood

Aurélie Truffot[a], Lara Cabezas[b], Manon Gnesotto[a], Julien Lupo[a], Patrice Morand[a], Thomas Jouve[b], Johan Noble[b], Raphaële Germi[a].

[a] Univ. Grenoble Alpes, CNRS, CEA, IRIG IBS, 38000 Grenoble, France

[b] Univ. Grenoble Alpes, CNRS, Inserm, CHU Grenoble Alpes IAB, 38000 Grenoble, France

Corresponding Author:

Dr Aurélie Truffot

ORCID: 0000-0002-7991-1733

E-mail address: atruffot@chu-grenoble.fr

Supplementary Materials and methods

Study population

Paired plasma and WB samples were collected from residual blood during routine clinical care in kidney transplant recipients between 01/2021 and 12/2022 in the Nephrology, Hemodialysis, Apheresis and Transplantation Department of Grenoble University Hospital (biological sample collection (CRB04 AC-2017-2949)). Samples were stored at −80 °C.

In this cohort, patients were initially treated by tacrolimus as immunosuppressive treatment and then converted to bélatacept. TTV DNA load was quantified in both matrices before the introduction of bélatacept and at three, six and twelve months after. Sixty-eight patients were included, with 218 samples.

All patients provided written informed consent for the conservation and reuse of their samples. The Grenoble University Hospital review board approved this retrospective monocentric study (registration RnIPH 2023, protocol SwitchAbela ; CNIL number: 2205066 v0). Biological (CMV, BKV, EBV load) and clinical data were collected from our medical database.

TTV DNA quantification

Viral DNA was extracted from 200 µL plasma or whole blood samples using the NucliSENS® easyMAG® automated system (bioMérieux, Marcy-l'Étoile, France), following the manufacturer's instructions. TTV DNA was quantified using the recently commercialized TTV R-gene® kit (bioMérieux, Marcy-l'Etoile, France). This quantitative real-time PCR (qPCR) assay targets a highly 128 pb conserved segment of the 5′ untranslated region of the viral genome with more than 90 % identity across isolates (18). Amplification detection and quantification were performed on LightCycler® 480 Instrument II (Roche Diagnostics, Manheim, Germany). The broad linear quantification range was from 250 to 10^9 copies/mL. This technique was developed for use with both plasma and whole blood.

Statistical Analysis

Continuous data were expressed as medians (25th–75th percentiles) and categorical variables as numbers (percentages). The correlation between TTV DNA loads in plasma and WB was evaluated using a Pearson's correlation test and R correspond to the correlation coefficient. Comparison of paired samples were performed using a Wilcoxon test. A 2-sided P-value of <0.05 was considered statistically significant. The Bland-Altman analysis was used to analyze the concordance and mean the difference between assays. The Cohen's kappa agreement test was used to analyze the concordance between positivity for TTV in plasma and that in WB. This method measures inter-observer agreement in qualitative category coding and the interpretation depend on the score (Landis *et al.,* Biometrics, 1977). Statistical analyses were conducted using the R statistical software (version 4.3.0; R Foundation for Statistical Computing, Vienna, Austria).

Supplementary Figures

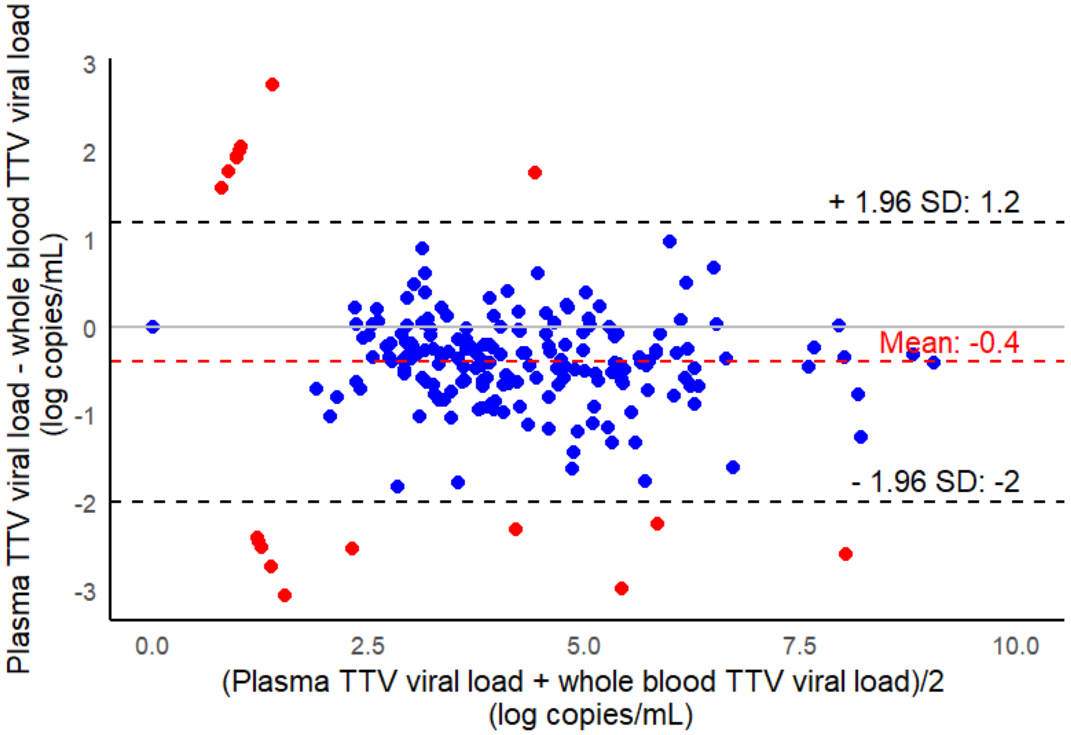

Figure S1: Bland-Altman representation of TTV viral loads obtained in plasma and whole blood. Horizontal lines are drawn at the mean difference (red line), and at the upper and lower limits of agreement (dashed lines). Outliers are presented in red. TTV, torquetenovirus; SD, standard deviation

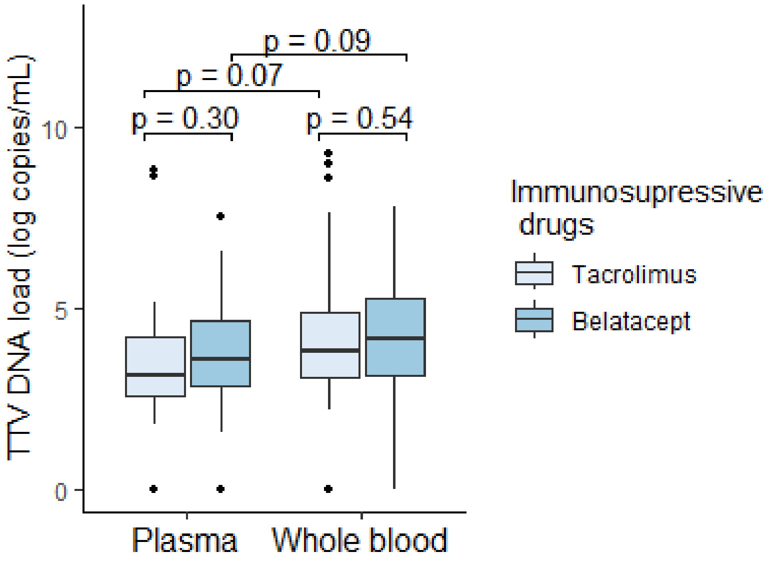

Figure S2: TTV DNA load performed at the same time in plasma and whole blood of patients on tacrolimus and then 12 months after conversion to belatacept. (Wilcoxon test, no significant difference, all *$p > 0.05$)*

Supplementary References

S1. Landis JR, Koch GG. The measurement of observer agreement for categorical data. Biometrics. mars 1977;33(1):159-74.

S2. Maggi F, Focosi D, Albani M, Lanini L, Vatteroni ML, Petrini M, et al. Role of Hematopoietic Cells in the Maintenance of Chronic Human Torquetenovirus Plasma Viremia. Journal of Virology. juill 2010;84(13):6891-3.

S3. Doberer K, Schiemann M, Strassl R, Haupenthal F, Dermuth F, Görzer I, et al. Torque teno virus for risk stratification of graft rejection and infection in kidney transplant recipients-A prospective observational trial. Am J Transplant. août 2020;20(8):2081-90.

S4. Haupenthal F, Rahn J, Maggi F, Gelas F, Bourgeois P, Hugo C, Jilma B, Böhmig GA, Herkner H, Wolzt M, Doberer K, Vossen M, Focosi D, Neuwirt H, Banas M, Banas B, Budde K, Viklicky O, Malvezzi P, Rostaing L, Rotmans JI, Bakker SJL, Eller K, Cejka D, Pérez AM, Rodriguez-Arias D, König F, Bond G; TTVguideTX consortium partners. A multicentre, patient- and assessor-blinded, non-inferiority, randomised and controlled phase II trial to compare standard and torque teno virus-guided immunosuppression in kidney transplant recipients in the first year after transplantation: TTVguideIT. Trials. 2023 Mar 22;24(1):213. doi: 10.1186/s13063-023-07216-0. PMID: 36949445; PMCID: PMC10032258.
